# A to Z of Health: An Evidence-Based Narrative Review of Multivitamin-Multimineral and Nutraceutical Supplementation

**DOI:** 10.7759/cureus.108032

**Published:** 2026-04-30

**Authors:** Vaidehi Nawathe, Shilpa Verma, Amitrajit Pal, Dattatray Pawar, Akhilesh Sharma

**Affiliations:** 1 Department of Dietetics and Applied Nutrition, Bhaktivedanta Hospital and Research Institute, Mumbai, IND; 2 Department of Medicine, Breach Candy Hospital, Sir H. N. Reliance Foundation Hospital, Mumbai, IND; 3 Department of Medical Affairs, Alkem Laboratories Ltd., Mumbai, IND

**Keywords:** deficiency, dietary supplements, minerals, nutraceuticals, vitamins

## Abstract

Multivitamin and mineral (MVM) supplements are dietary products that contain a combination of essential vitamins and minerals, and sometimes additional bioactive compounds. They are used to meet daily nutrient requirements, support physiological functions, and address increased nutritional needs, including in special populations. This narrative review summarizes the physiological roles, dietary sources, and deficiency symptoms of essential vitamins and minerals. Additionally, recommended dietary allowances (RDAs) and tolerable upper intake levels (ULs) of vitamins and minerals according to the Indian Council of Medical Research-National Institute of Nutrition (ICMR-NIN) are included. The review included details from various clinical studies, including randomized controlled trials, meta-analyses, and cohort studies on MVM supplementation, with special emphasis on cardiovascular, endocrine (especially diabetic mellitus), cancer, cognitive, skeletal, immune, and ocular health outcomes. It also highlights evidence in special populations such as pregnant and lactating women, infants, children, adolescents, older adults, and athletes. Furthermore, evidence related to nutraceuticals such as omega-3 fatty acids, lutein, curcumin, ginseng, brahmi, ashwagandha, and coenzyme Q10 is included. The review covers the safety and toxicity aspects of MVM supplements; however, limited evidence is available on drug-nutrient interactions. Overall, MVM supplements, along with nutraceuticals, may play a valuable role in targeted health optimization, especially in populations at risk of deficiencies. Standardized formulations, long-term safety evaluation, and integration of personalized nutrition approaches are critical to enhancing their clinical and public health impact.

## Introduction and background

More than two billion people are at risk of micronutrient deficiencies, which often involve multiple, rather than single, nutrients [1,2]. The most common deficiencies include vitamin A, folic acid, vitamin D, iron, iodine, and zinc, especially among children and women in low- and middle-income countries [3-5]. Even in high-income countries, a substantial proportion of older adults are micronutrient-deficient [6]. In India, nutritional deficiencies contributed to 0.5% of all deaths in 2016 [7]. Further, a study in the general population of India reported a high prevalence of iron (54%), vitamin B-12 (53%), and folic acid (37%) deficiencies, with iron deficiency reaching 61% among pregnant women [8]. Among school children and adolescents, deficiencies of calcium (59.9%), iron (49.4%), vitamin D (39.7%), and vitamin B-12 (33.4%) were reported [9].

Considering the high prevalence of micronutrient deficiencies across diverse populations, effective strategies are needed to address these nutritional gaps. Multivitamin and mineral (MVM) supplementation offers a practical approach to improving micronutrient intake. MVM supplements are dietary products that contain a combination of essential vitamins and minerals, and sometimes additional nutraceuticals in a single formulation. They are widely used to help individuals meet their daily nutrient requirements, support overall health, and address increased nutritional needs associated with pregnancy, aging, and illness. However, they are not substitutes for a balanced diet rich in whole foods, which provide fiber and other beneficial compounds [10].

The use of MVM supplements is widespread in the USA and other developed countries. According to the National Health and Nutrition Examination Survey, MVM use in the USA increased from 35% in 1999-2000 [11] to 49-52% between 2011 and 2014, with higher usage among women than men [12-14]. In pregnant women, reported usage ranges from 78% to 97.5% in the USA, Canada, and Australia [15-17]. In India, the use of dietary supplements is increasing at a rate of 25% [18].

A clinical study evaluated the role of MVM supplementation in improving nutritional status and delaying the progression of certain chronic diseases [19]. Large-scale studies have reported modest but significant benefits of long-term MVM supplementation in reducing cardiovascular disease (CVD), diabetes mellitus, and cancer incidence, and improving neurological and bone health [20-27]. Furthermore, clinical studies including special populations have reported that MVM supplementation is associated with improved maternal and infant health, infertility and post-menopausal symptoms, enhanced growth and academic performance in children and adolescents, support for cognitive and psychosocial function in older adults, and improved recovery in athletes [28-36].

In this narrative review, we have provided a comprehensive summary of the current clinical evidence for the efficacy and safety of MVM supplementation in preventing and managing chronic diseases, including findings in both general and special populations. We also outline key food sources of essential vitamins, minerals, and nutraceuticals, along with their physiological roles (function in the body), recommended dietary allowances (RDAs), and tolerable upper intake levels (ULs), to guide the safe and effective consumption of MVM supplements.

## Review

Methods

A comprehensive literature search was conducted using PubMed and Google Scholar. The search included following Medical Subject Headings (MeSH) terms and keywords: “multivitamin”, “minerals,” “physiological,” “chronic disease,” “diabetes mellitus,” “cardiovascular diseases,” “neoplasms,” “cognition,” “bone,” “immune system,” “eye diseases,” “pregnant”, “infants,” “adolescent,” “aged,” and selected nutraceuticals including “Fatty acids, omega-3,” “lutein,” “curcumin,” “panax ginseng,” “brahmi,” and “coenzyme Q10,” “alpha-lipoic acid,” “amino acids,” “ashwagandha,” “astaxanthin,” “carnitine,” “choline,” “citrus,” “docosahexaenoic acids,” “evening primrose oil,” “inositol,” “theanine,” “lycopene,” “piperine,” “resveratrol,” zeaxanthin.” Terms were used individually and in combination with Boolean operators (AND/OR).

Articles published in English were screened based on titles and abstracts, followed by full-text review for eligibility. The review synthesizes findings from previously published studies without conducting any meta-analysis or statistical pooling. Studies were selected based on their relevance and quality to provide an overview of the current evidence, with priority given to randomized controlled trials (RCTs), clinical studies, systematic reviews, and meta-analyses. Additional studies were identified through manual screening of reference lists.

Micronutrients and nutraceuticals: biological functions, dietary sources, and health implications

Vitamins and Minerals

Vitamins and minerals play essential roles in a variety of physiological functions within the body. They are involved in key metabolic pathways, including energy-yielding metabolism, DNA synthesis, oxygen transport, and neuronal signaling, all of which are important for maintaining cellular function [37]. A wide variety of foods provide the vitamins and minerals necessary for optimal health, such as fruits, vegetables, whole grains, legumes, dairy products, meats, seafood, nuts, and seeds. Each of these contributes distinct micronutrients in varying amounts; hence, including a diverse range of these foods in the daily diet helps ensure an adequate intake of micronutrients and reduces the risk of deficiencies [38]. The key physiological roles, common dietary sources, and primary deficiency symptoms of various vitamins and minerals are summarized in Table 1.

**Table 1:** Key physiological roles, common dietary sources, and primary deficiency symptoms of various vitamins and minerals † Although vitamins D2 and D3 are two different forms, both function as prohormones and are used by the body in an identical manner. The only difference lies in the structure of their side chains. ‡ Not considered essential by some authorities. § Primary deficiency symptoms are extremely rare. BP: blood pressure; CNS: central nervous system; DNA: deoxyribonucleic acid

Micronutrients	Key physiological roles	Common dietary sources	Primary deficiency symptoms
Vitamin A [39]	Vision in darkness, cornea and conjunctiva development, immune system functioning, bone and fetus development, CNS formation	Carrots, sweet potatoes, spinach, tomato, papaya, red peppers, loquat, mango, apricots, cheese, milk, cereals, beef liver, fish, egg yolks	Impaired vision, weight loss, impaired immune responses, and increased susceptibility to infections, impaired spermatogenesis, anemia, loss of taste, and smell sensations
Vitamin B-1 (Thiamine) [40-43]	Energy production, neurotransmitter synthesis, neuronal signal transmission, myelin synthesis, antioxidant	Whole grains, fortified bread/cereals/pasta/brown rice, nuts, black beans, eggs, pork, fish, liver	Fatigue, weight/appetite loss, confusion, memory loss, muscle weakness, confabulation, cardiovascular dysfunction, peripheral nerve damage (beriberi), tingling sensations/numbness (Wernicke-Korsakoff syndrome)
Vitamin B-2 (Riboflavin) [42,44]	Energy production, cellular function, growth and development, metabolism of fats, drugs, and steroids, and homocysteine regulation	Spinach, fortified cereals/grains, milk, mushrooms, organ meat (kidneys, liver)	Skin disorders, sores at mouth corners, swollen/cracked lips, hair loss, sore throat, reproductive/nervous system problems, anemia, malabsorption
Vitamin B-3 (Niacin) [40,42,45,46]	Energy conversion, cell development/function	Nuts, grains, cereals, mushrooms, legumes, meat, fish	Pellagra (dermatitis/photo dermatitis, alopecia, muscle weakness, twitching/burning in the extremities, altered gait, and diarrhea), neurological symptoms such as depression, anxiety, vertigo, memory loss, paranoia, psychosis, and aggression
Vitamin B-5 (Pantothenic Acid) [42,47]	Energy production, synthesis/breakdown of fats, precursor for acetyl-CoA synthesis	Avocados, potatoes, broccoli, whole grains, peanuts, sunflower seeds, chickpeas, eggs, milk, mushrooms, and organ meats	Numbness/burning sensations in extremities, dermatitis, headache, fatigue, irritability, restlessness, sleep disturbances, stomach pain, heartburn, diarrhea, nausea, vomiting, appetite loss
Vitamin B-6 (Pyridoxine) [42,48,49]	Protein metabolism, brain development (pregnancy/infancy), immune function, neurotransmitter biosynthesis, and homocysteine regulation	Potatoes, starchy vegetables, banana, non-citrus fruits, chickpeas, poultry, fish, organ meats, beef liver, salmon	Anemia, depression, confusion, weakened immune system, irritability, sensitive hearing
Vitamin B-7 (Biotin) [50,51]	Convert carbohydrates, fats, and proteins into energy, cofactors for carboxylases, gene regulation, cell signaling, development of hair, skin, and nails	Seeds, nuts, sweet potatoes, spinach, broccoli, peanuts, soyabeans, milk, mushrooms, organ meats, eggs, fish, meat	Hair thinning/loss, skin rashes around eyes, nose, mouth, and anal area, styes, high acid in blood/urine, seizures, skin infection, brittle nails, depression, lethargy, hallucinations, weak muscle tone, developmental delay in infants
Vitamin B-9 (Folate/Folic Acid) [40,42,52,53]	DNA/genetic material synthesis, cell division, cerebral methylation, neuronal/glial membrane lipids	Asparagus, brussels sprouts, spinach, nuts, beans, peas, fortified bread, flour, cereals, beef liver	Megaloblastic anemia (weakness, fatigue, lack of concentration), mouth/tongue sores, skin/hair/fingernail color changes, neural tube defects
Vitamin B-12 (Cobalamins) [40,42,52,54]	Blood/nerve cell health, DNA production, CNS development, nerve myelination, homocysteine conversion	Fortified cereals, milk, dairy, eggs, fish, meat, poultry, clams, oysters, beef liver, nutritional yeasts	Megaloblastic anemia (weakness, fatigue, lack of concentration), loss of balance, depression, confusion, dementia, poor memory
Vitamin C (Ascorbic Acid) [40,55]	Antioxidant, collagen production, iron absorption (plant-based), immune system support, neurotransmitter modulation	Kakadu plum, star fruit, guava, black currant, citrus fruits (oranges, grapefruit), red/green peppers, kiwifruit, strawberries, broccoli, kale, peppers, potatoes, tomatoes, rose hips, sea buckthorn, chives, parsley	Scurvy, weakness, swelling, fatigue, gum inflammation, petechiae, joint pain, poor wound healing, bleeding gums, tooth loss
Vitamin D^†^ (Calciferol) [48,56]	Calcium homeostasis/absorption, bone mineralization, keratinocyte growth, remodeling, muscle movement, nerve communication, immune system support	Fortified milk, cereals, juices, yogurt, cheese, margarine, egg yolks, fatty fish (trout, salmon, tuna, mackerel), fish liver oils, tuna liver oil, cod liver oil, beef liver, tilapia, herring, sun exposure, UV-irradiation	Rickets in children, osteomalacia or osteoporosis in teens and adults, bone pain, muscle weakness, and fatigue in breastfed infants, older adults, and those with limited sun exposure, dark skin, fat malabsorption, increased risk of obesity, hypertension, type 1 diabetes, multiple sclerosis, cancer, renal disorders, and gastrointestinal disorders
Vitamin E (Tocopherol) [57-60]	Antioxidant, immune function, cell signaling, dilatation of blood vessels, and prevention of clotting	Vegetable oils (wheat germ, sunflower, safflower, corn, soybean), nuts (peanuts, hazelnuts, almonds), seeds (sunflower), spinach, broccoli, kiwifruit, mango, tomato, fortified cereals, juices, margarines	Nerve/muscle damage, loss of sensations in extremities, impaired motor control, muscle weakness, vision problems, weakened immune system
Vitamin K (Phylloquinone) [61]	Blood clotting, bone and joint health, bone mineralization, inhibition of vascular stiffness, improvement of endothelial function, maintenance of strong teeth, brain development, optimal body weight	Leafy vegetables, plant oils	Bleeding, impaired blood clotting, kidney disorders, bone health, and bone fracture risk
Vitamin K2 (MK-7) (Menaquinone) [62,63]	Essential for bone strength, blood vessel health, and brain development	Natto (fermented soybean), animal products (meat, dairy)	Bleeding disorder, kidney disorders, and osteoporosis risk
Calcium [64,65]	Building and maintaining healthy bones and teeth, muscle contraction, nerve signal transmission, blood clotting, regulation of the immune system, BP, body fluids, and hormones	Milk, yogurt, cheese, orange juice, nuts, sesame, chia seeds, watercress, kale, broccoli, fortified juices, milk substitutes, tofu, canned sardines/salmon (with bones)	Weak/fragile bones, osteomalacia, osteoporosis
Magnesium [64,66]	Muscle contraction, nerve signal transmission, blood sugar and BP regulation, maintaining heart rhythm, energy production, and active transport of calcium/potassium	Legumes, cashew nuts, peanuts, pumpkin seeds, chia seeds, almonds, whole grains, green leafy vegetables (boiled spinach), fortified cereals, soy milk, baked potato, black beans cooked, brown rice	Alzheimer’s disease, attention deficit hyperactivity disorder, insulin resistance, hypertension, migraine headaches, osteoporosis, loss of appetite, fatigue, weakness, numbness, tingling sensations, seizures, personality changes (increased irritability, anxiety, and depression), abnormal heart rhythm
Potassium [64]	Nerve signal transmission, skeletal and smooth muscle contraction, digestive and kidney functions, cellular tonicity, and intracellular fluid volume regulation	Fruits/vegetables, legumes, canned kidney beans, orange juice, banana, boiled soybeans, baked potatoes, whole-wheat flour, brown rice, dried apricot, cooked lentils, dried prunes, mashed squash, raisins	Hyperkalemia (irritability, nausea, urine excretion), cardiac conduction disturbances, neuromuscular dysfunction, muscle weakness, acne, dry skin, nervous disorders, irregular heartbeat, loss of gastrointestinal tone, cardiac arrhythmia, increased blood sugar
Chromium^‡ ^[65]	Metabolism of fats and carbohydrates, cognitive processes, stimulation of fatty acids and cholesterol synthesis, insulin and glucose regulation	Whole grains, wheat germ, nuts, green peppers, apples, bananas, spinach, black pepper, cheese, butter, molasses, beef, liver, eggs, chicken, oysters, brewer's yeast, unrefined foods	No deficiency reported in healthy individuals
Copper [40,65]	Energy production, promotes healing, blood vessel formation, nervous/immune system maintenance, gene activation, brain development, iron homeostasis	Nuts (cashews), seeds (sesame), whole-grain products, avocados, greens (broccoli, mustard greens), legumes, milk and dairy products, fortified tofu and fortified soy milk, canned fish with bones (salmon, sardines)	Extreme tiredness, lightened skin patches, weak/brittle bones
Iodine [65,67]	Production of thyroid hormones, blood cell production, bone and brain development (pregnancy/infancy)	Iodized salt, bananas, cranberries, dried prunes, strawberries, dairy (milk, yogurt, cheese), eggs, seafood (cod fish, shrimp, lobster), baked turkey breast, canned tuna, canned corn	Inadequate thyroid hormone production, stunted growth, intellectual disability, delayed sexual development, impaired mental development (children), reduced cognitive function (adults), goiter
Iron [48,65,68]	Hemoglobin/myoglobin production (oxygen transport), growth, development, cell function, hormone synthesis	Fortified cereals/breads, white beans, lentils, pumpkin, squash seeds, spinach, kidney beans and pulses, peas, nuts (cashew, pine, hazelnuts, almond), dried fruits (nonheme iron), lean meat, seafood, poultry (heme iron)	Gastrointestinal upset, weakness, tiredness, lack of energy, concentration/memory problems, impaired immunity, exercise capacity, and temperature control, learning difficulties (children), anemia
Manganese [65,69]	Cofactor for enzyme function, brain function, contributes to the health of the nervous system, bone formation, carbohydrate, amino acid, and cholesterol metabolism, maintains bone health in postmenopausal women	Legumes, nuts, seeds, beans, whole grains, leafy green vegetables, tea, bread, tofu, fish, seafood	No deficiency reported in healthy individuals, but theoretical symptoms include impaired growth, skeletal abnormalities, impaired carbohydrate and fat metabolism
Molybdenum^§ ^[65,70]	Cofactors for several enzymes involved in protein processing, drug and toxin breakdown, components of various enzymes (sulfite oxidase, xanthine oxidase, aldehyde oxidase)	Legumes (black-eyed peas, lima beans), whole grains, nuts, leafy vegetables, chard, sunflower seeds, wheat flour, cucumber, dairy, eggs, pasta, breads	Molybdenum cofactor deficiency (seizures, brain damage; often fatal)
Selenium [65,71]	Antioxidant, development and function of immune cells, thyroid hormone metabolism, DNA synthesis, reproductive health	Leafy and green vegetables, cereals, whole grains, dairy products, bread, Brazil nuts, seafood, organ meats, muscle meats	Keshan disease (cardiomyopathy), Kashin-Beck disease (osteoarthropathy), impaired immunity, male infertility, muscle weakness, hair loss, skin/nail changes
Zinc [40,65]	Immune system support, DNA/protein production, growth/development (pregnancy, infancy, childhood, adolescence), wound healing, taste/smell detection	Leavened whole grain, fortified breakfast cereals, beans, nuts, whole grains, eggs, dairy products, oysters, meat, fish, poultry, seafood (crab, lobsters)	Diarrhea, appetite loss (infants/children), reproductive problems (adulthood), hair loss, frequent infections and slow recovery, impaired taste/smell, delayed wound healing, cognitive issues (older adults)

Nutrient requirements vary significantly across the stages of life, influenced by factors such as growth rate, physiological changes, metabolic demands, and special conditions such as pregnancy and lactation [72]. These requirements are often expressed as RDAs or Dietary Reference Intakes. RDAs specify the intake required for different age groups, sexes, and physiological conditions to meet the nutritional needs of 97%-98% of healthy individuals (NRD). In India, RDAs are established by the Indian Council of Medical Research (ICMR)-National Institute of Nutrition (NIN), based on scientific evidence relevant to the nutritional needs of the Indian population [73]. A summary of the daily requirements for key vitamins and minerals according to ICMR-NIN 2024 is provided in Table 2.

**Table 2:** RDAs of key vitamins and minerals for the Indian population according to ICMR-NIN 2024 Included values available in the ICMR short summary report of nutrient requirements for Indians. † Adequate intake (AI). ‡ Range indicates RDA for sedentary to active individuals. § For 0-6 months. ¶ For 7-12 months. B: boys; G: girls; ICMR-NIN: Indian Council of Medical Research-National Institute of Nutrition; M: men; RDA: recommended dietary allowance; W: women

Nutrients	Infants (0-6 months^†^)	Infants (6-12 months)	Children (1-3 years)	Children (4-6 years)	Children (7-9 years)	Adolescence (10-12 years)	Adolescence (13-15 years)	Adolescence (16-18 years)	Adults	Pregnancy	Lactation
Vitamin A (µg/day)	350	350	390	510	630	770 (B) 790 (G)	930 (B) 890 (G)	1000 (B) 860 (G)	1000 (M) 840 (W)	900	950
Vitamin B-1 (mg/day)	0.2	0.4	0.7	0.9	1.1	1.5 (B) 1.4 (G)	1.9 (B) 1.6 (G)	2.2 (B) 1.7 (G)	1.4-2.3^‡^ (M) 1.4-2.2^‡^ (W)	2.0	2.1
Vitamin B-2 (mg/day)	0.4	0.6	1.1	1.3	1.6	2.1 (B) 1.9 (G)	2.7 (B) 2.2 (G)	3.1 (B) 2.3 (G)	2.0-3.2^‡^ (M) 1.9-3.1^‡^ (W)	2.7	3.0^§^ 2.9^¶^
Vitamin B-3 (mg/day)	2	5	7	9	11	15 (B) 14 (G)	19 (B) 16 (G)	22 (B) 17 (G)	14-23^‡^ (M) 11-18^‡^ (W)	13	16
Vitamin B-6 (mg/day)	0.1	0.6	0.9	1.2	1.5	2.0 (B) 1.9 (G)	2.6 (B) 2.2 (G)	3.0 (B) 2.3 (G)	1.9-3.1^‡^ (M) 1.9-2.4^‡^ (W)	2.3	2.16^§^ 2.07^¶^
Vitamin B-9 (µg/day)	25	85	120	135	170	220 (B) 225 (G)	285 (B) 245 (G)	340 (B) 270 (G)	300 (M) 220 (W)	570	330
Vitamin B-12 (µg/day)	1.2	1.2	1.2	2.2	2.2	2.2	2.2	2.2	2.2	2.45	3.2
Vitamin C (mg/day)	20	30	30	35	45	55 (B) 50 (G)	70 (B) 65 (G)	85 (B) 70 (G)	80 (M) 65 (W)	80	115
Vitamin D (IU/day)	400	400	600	600	600	600	600	600	600	600	600
Calcium (mg/day)	300	300	500	550	650	850	1000	1050	1000	1000	1200
Magnesium (mg/day)	30	75	90	125	175	240 (B) 250 (G)	345 (B) 340 (G)	440 (B) 380 (G)	440(M) 370 (W)	440	400
Iodine (µg/day)	100	130	90	90	90	100	140	140	140	220	280
Iron (mg/day)	-	3	8	11	15	16 (B) 28 (G)	22 (B) 30 (G)	26 (B) 32 (G)	19 (M) 29 (W)	27	23
Zinc (mg/day)	-	2.5	3.3	4.5	5.9	8.5	14.3 (B) 12.8 (G)	17.6 (B) 14.2 (G)	17 (M) 13.2 (W)	14.5	14.1

In addition to RDAs, ULs are critical in ensuring nutrient safety [74]. ULs represent the highest daily intake from all sources over the long-term without causing adverse effects [75,76]. These values guide safe supplementation practices and help to prevent excessive intake [74]. The ULs for essential nutrients are provided in Table 3.

**Table 3:** ULs of key vitamins and minerals for the Indian population according to ICMR-NIN 2024 Values available in the ICMR short summary report of nutrient requirements for Indians were included. † ULs values are only for non-dietary pharmacological doses. ULs for vitamin B-1, vitamin B-2, vitamin B-5, vitamin B-7, vitamin B-12, vitamin K, potassium, and chromium are not established either due to no reported adverse events at high doses or due to insufficient data on adverse effects. B: boys; G: girls; ICMR-NIN: Indian Council of Medical Research-National Institute of Nutrition; RE: retinol equivalent; UL: tolerable upper intake level

Nutrients	Infants (0-6 months)	Infants (6-12 months)	Children (1-3 years)	Children (4-6 years)	Children (7-9 years)	Adolescence (10-12 years)	Adolescence (13-15 years)	Adolescence (16-18 years)	Adults	Pregnancy	Lactation
Vitamin A (µg/day)	600	600	600	900	900	1700	2800	2800	3000	3000	3000
Vitamin B-3 (mg/day)	-	-	-	-	-	-	-	-	35	-	-
Vitamin B-6 (mg/day)	-	-	-	-	-	-	-	-	100	-	-
Vitamin B-9 (µg/day)	-	-	-	-	300	600-800	600-800	600-800	1000	1000	1000
Vitamin C (mg/day)	-	-	350	550	800	1050 (B) 1300 (G)	1550 (B) 1800 (G)	1950 (B) 2000 (G)	2000	2000	2000
Vitamin D (IU/day)	1000	1500	2500	3000	3000	4000	4000	4000	4000	4000	4000
Calcium (mg/day)	-	-	1500	2500	2500	3000	3000	3000	2500	2500	2500
Magnesium^†^ (mg/day)	-	-	65	110	110	350	350	350	350	350	350
Iodine (µg/day)	-	-	200	300	400	600	900	1100	1100	1100	1100
Iron (mg/day)	40	40	40	40	40	40	45	45	45	45	45
Zinc (mg/day)	4	5	7	12	12	23	34	34	40	40	40

Nutraceuticals

**Figure 1:**
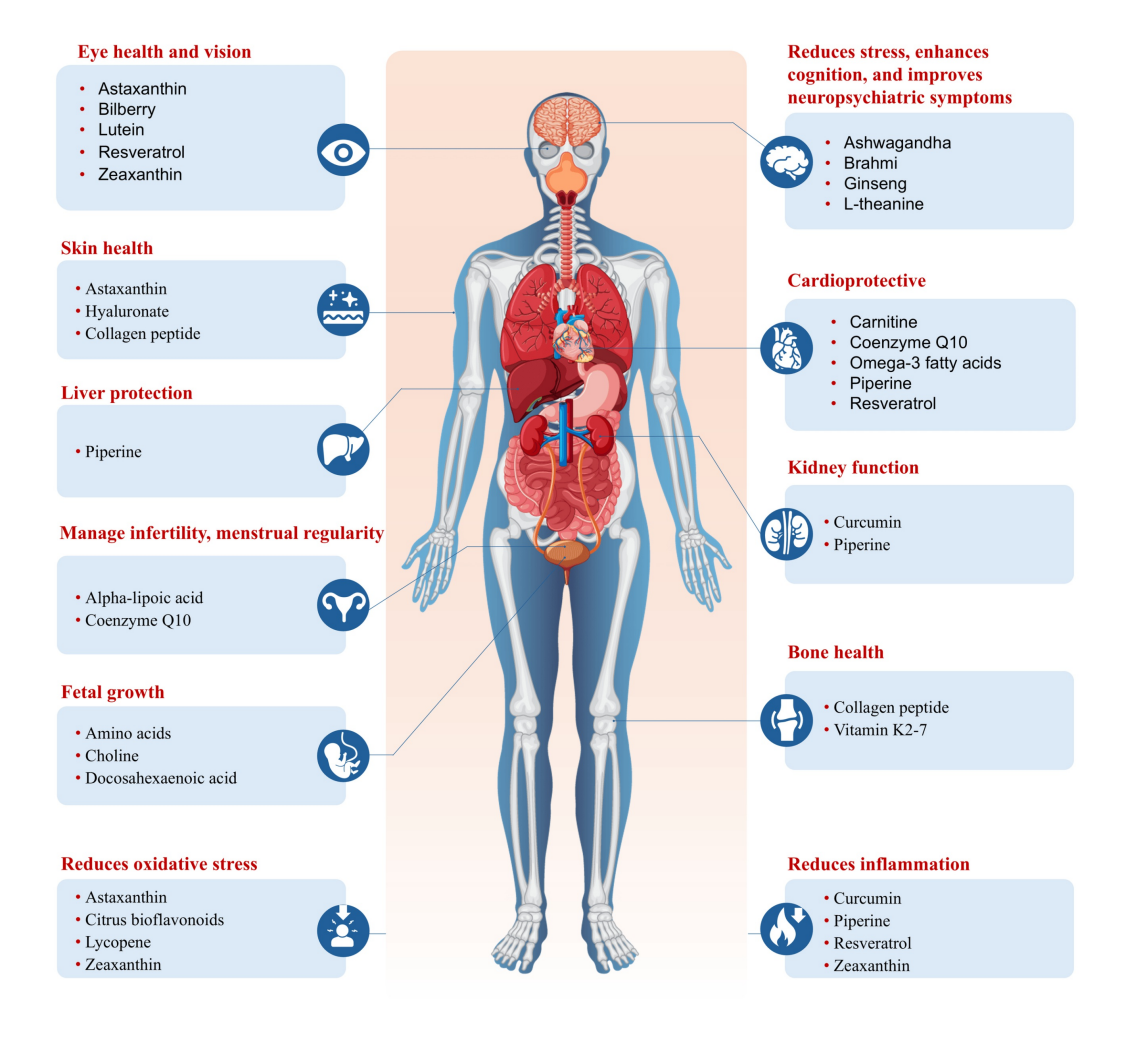
Beneficial effects of nutraceuticals on health Note: Refer to Table 4 for detailed information, including source citations, on the beneficial effects of nutraceuticals. Image credit: This is an original image created by the authors using Adobe Illustrator (Adobe Inc., San Jose, CA, USA; version 2025), and no AI tools were used.

Nutraceuticals are bioactive compounds derived from foods that possess therapeutic potential [77]. They may function as antioxidants, anti-inflammatories, lipid-lowering agents, immunomodulators, neuroprotective agents, or metabolic regulators, and are increasingly recognized as valuable adjuncts in disease prevention and management [78,79]. The key sources and beneficial effects of various nutraceuticals are represented in Table 4 and Figure 1.

**Table 4:** Beneficial effects and sources of nutraceuticals

Nutraceuticals	Beneficial role	Sources
Alpha-lipoic acid [80]	Improves and prevents diabetic neuropathy and reduces lipid peroxidation indices	Organ meat, spinach, broccoli, tomatoes, peas, brussels sprouts, rice
Amino acids [81]	Support fetal growth	Animal meat, vegetables, alanine, glutamine
Ashwagandha [82]	Reduces cortisol level, improves stress and anxiety	Withania somnifera (plant)
Astaxanthin [83-85]	Skin and eye health	Algae, yeast, salmon, trout, krill, shrimp, crayfish
Bilberry [86]	Improves night vision	Vaccinium myrtillus (plant)
Brahmi [87,88]	Enhances memory and attention	Bacopa monnieri (plant)
Carnitine [89-92]	Lowers the risk of heart attack and manages men's infertility	Alfalfa, avocado, cauliflower, wheat, bread, beef (liver, kidney, heart), chicken, lamb, cow’s milk, casein, peanuts, torula yeast
Choline [90,93,94]	Helps in brain development in the fetus and children	Beef liver, chicken liver, eggs, wheat germ, bacon, raw soyabean, pork, apples, avocados, tomatoes, carrots, onions
Citrus bioflavonoids [95,96]	Regulate oxidative stress	Citrus fruits
Coenzyme Q10 (Ubiquinones) [97]	Reduces the risk of mortality in heart failure	Meat, fish, nuts, oils
Curcumin [98,99]	Anti-inflammatory effects	Turmeric
Docosahexaenoic acid [100,101]	Supports neurodevelopment and vision	Seafoods, salmon, mackerel, herring, trout, fish oil, krill oil, algal oil, flaxseed oil, perilla oil, human breastmilk
Evening primrose oil (Oenothera biennis) [102]	Maintain skin health and integrity	Oenothera biennis (plant)
Ginseng [103,104]	Neuroprotection and improves cognition	Gingko biloba (plant)
Inositol [105-108]	Improves menstrual regularity and ovulation	Bran, seeds, cereals, legumes, oil, nuts
L-theanine [109]	Supports relaxation and attention	Black tea, green tea, white tea, oolong, pu-erh
Lutein [110]	Supports macular pigment and eye health	Kale, spinach, broccoli, peas, lettuce, egg yolk, einkorn, Khorasan, durum wheat, corn
Lycopene [111,112]	Antioxidant effects	Tomatoes, pink guavas, apricots, watermelons, pink grapefruit
Omega-3 fatty acids [48,113-115]	Anti-inflammatory effects, cardioprotective, improve insulin sensitization, and bone protection	Fatty fish, fish oil (anchovies, sardines, herring, caplin), soybean, canola oils, flax seeds, chia seeds, echium oil, nuts
Piperine [116]	Liver protective, cardioprotective, and inhibits inflammation	Black pepper, long pepper
Resveratrol [117,118]	Regulates inflammatory response, lipid catabolism, supports cellular detoxification and recycling	Red wine, peanuts, apples, fresh grapes, mulberries, lingonberries, cranberries, red currants, bilberries, pistachios, red grape juice
Zeaxanthin [119,120]	Maintains and improves visual function, antioxidant, anti-inflammatory, regulates liver and kidney function	Kale, spinach, lettuce, broccoli, corn, carrot, cabbage, melon, zucchini, peppers, gooseberries, kiwi, blackcurrants, avocados, raspberries, goji berries, egg yolk

Clinical evidence for the role of MVM and nutraceutical supplementation in supporting nutrient requirements and preventing chronic diseases

Dietary supplements are intended to complement the diet, not to prevent or treat disease [121]. However, long-term use of MVM supplements and nutraceuticals may contribute to the prevention or delayed progression of certain chronic diseases [19]. This section provides an overview of the current clinical evidence regarding their role in supporting nutrient adequacy, promoting overall health, and reducing the risk of chronic diseases.

Nutritional Requirements and General Well-Being

MVM supplementation increases nutrient intake and helps achieve the recommended requirements of vitamins and minerals when these needs are not met from food alone [10]. A double-blind, placebo-controlled trial demonstrated that supplementation with B-complex vitamins, brahmi, and ginseng improved nutrient biomarkers in healthy, middle-aged adults who were consuming an optimal diet prior to supplementation [122]. Moreover, a previous review highlighted the beneficial effects of various micronutrients (B-complex vitamins, vitamin C, iron, magnesium, and zinc) in enhancing well-being (reducing perceived mental and physical fatigue) and promoting a positive mood [37].

Cardiovascular Diseases (CVDs)

Several clinical studies, including meta-analyses [27,123-125], RCTs [126,127], and a cohort study [128], have evaluated the potential benefits of MVM supplementation in reducing the mortality risk associated with CVD. Meta-analyses, systematic reviews, and RCTs found no significant effect of multivitamins and minerals (calcium, selenium, zinc, and vitamin A) on CVD risk or all-cause mortality. Nonetheless, specific nutrients such as folic acid and B-complex vitamins have been associated with a reduced risk of stroke (risk ratio (RR): 0.79; p = 0.002 and 0.90; p = 0.04, respectively) [129,130]. Similarly, a recent meta-analysis showed that B-complex vitamin combinations significantly reduced stroke (RR: 0.79; 95% CI 0.68 to 0.93) and major adverse cardiovascular events (MACE; 0.80; 95% CI 0.69 to 0.92) in participants without pre-existing CVD and a modest benefit for MACE (0.91; 95% CI 0.83 to 0.99) in participants with established CVD. Overall, effects remain inconsistent, suggesting benefits may be population-specific [125]. The Women’s Health Study, a large cohort study involving 37,193 women with a mean follow-up of 16.2 years, reported no association between multivitamin use and major cardiovascular events [131]. In contrast, a large prospective study (n = 465,278) using UK Biobank data reported that MVM supplementation was associated with a modest reduction in cardiovascular events (p < 0.05) [132]. Similarly, the Physicians’ Health Cohort Study I, which followed 18,530 men physicians aged ≥40 years with a mean follow-up of 12.2 years, reported a lower risk of CVD among long-term MVM users (≥20 years) (hazard ratio (HR): 0.56; 95% CI 0.35 to 0.90; p = 0.05) [128]. Overall, the evidence demonstrated that MVM supplementation for CVD prevention lacks strong support and remains inconclusive.

On the other hand, various nutraceuticals have shown more consistent cardiovascular benefits. An umbrella review of 83 meta-analyses demonstrated a statistically significant effect of omega-3 fatty acid supplements in reducing total mortality from CVD (RR: 0.92; 95% CI 0.86 to 0.98) [133]. Similarly, a meta-analysis of 15 RCTs found that omega-3 fatty acids reduced cardiovascular events (RR: 0.95; 95% CI 0.91 to 0.99; p = 0.026) and mortality (RR: 0.94; 95% CI 0.88 to 0.99; p = 0.028) [134]. Further, a meta-analysis of 14 RCTs including patients with heart failure receiving coenzyme Q10 supplements showed a reduction in mortality (RR: 0.69; 95% CI 0.50 to 0.95; p = 0.02) and improvement in exercise capacity (standardized mean difference (SMD): 0.62; 95% CI 0.02 to 0.30; p = 0.04) [135]. The efficacy of curcumin in lowering lipid levels in patients with cardiovascular risk factors was also reported [136]. However, no beneficial effect of ginseng was observed in reducing CVD mortality, cardiovascular events, and peripheral arterial disease [137,138].

Cancers

According to the United States Preventive Services Task Force, there is inadequate evidence to support the use of individual vitamins, minerals, or nutraceuticals for cancer prevention in healthy populations without known nutritional deficiencies [139]. Among cancer survivors who received MVM supplementation, a lower total cancer mortality rate was reported [140]. A recent meta-analysis involving 28,558 men and 20,542 women reported a reduction in cancer incidence among men after MVM supplementation (fixed effect HR: 0.91; 95% CI 0.85 to 0.97; random effect HR: 0.88; 95% CI 0.77 to 1.01) [141]. However, the COcoa Supplement and Multivitamin Outcomes (COSMO) trial and the Selenium and Vitamin E Cancer Prevention Trial (SELECT) reported no beneficial effects of MVM supplements in patients with cancers [23,126].

Furthermore, omega-3 fatty acid supplementation has shown no significant effect on cancer incidence or overall mortality, as per a meta-analysis of RCTs by Zhang and colleagues [142]. In contrast, short-term curcumin supplementation has demonstrated a reduction in aberrant crypt foci, suggesting a potential preventive role in early colorectal changes [143].

Diabetes Mellitus

In patients with diabetes and micronutrient deficiencies, MVM supplementation demonstrated better glycemic control [21]. A small pilot crossover study reported that daily MVM supplementation for three months improved the well-being in patients with type 2 diabetes (p = 0.021), even in the absence of significant metabolic improvements [144]. Among patients with diabetes and with high fasting blood sugar levels, zinc and MVM supplementation demonstrated better glycemic control (mean reduction of fasting blood sugar levels -0.33 mmol/L, p = 0.05; reduction of HbA1c -0.01 %, p < 0.001) [22]. Further, a U.S. cohort study involving 232,007 patients showed the use of MVM supplements was associated with a significantly lower risk of developing diabetes (odds ratio (OR): 0.85; 95% CI 0.80 to 0.90) [145].

Further, an RCT demonstrated that 12 weeks of nutraceutical supplementation (bitter melon, fenugreek, cinnamon, alpha-lipoic acid, zinc, biotin, chromium, and cholecalciferol) led to improvements in glycemic control and insulin resistance [146]. A double-blind, randomized, placebo-controlled trial including 82 obese patients with type 2 diabetes showed a significant reduction in glucose (135.7 ± 19.5 mg/dL vs. 126.5 ± 16.8 mg/dL, p < 0.05) and HbA1c (8.3 ± 0.3% vs. 6.03 ± 0.58%, p < 0.05) levels after alpha-lipoic acid, carnosine, and vitamin B-1 supplementation [147]. A meta-analysis including 30 RCTs demonstrated the protective effects of omega-3 fatty acid supplements with significant reduction in fasting blood glucose (SMD: -0.48; 95% CI -0.76 to -0.19; p = 0.01) and insulin resistance (SMD: -0.61; 95% CI -0.98 to -0.24; p = 0.01) [148]. A meta-analysis of 40 RCTs reported the beneficial effects of coenzyme Q10, with significant effect on fasting glucose (weighted mean difference (WMD): -5.22 mg/dL; 95% CI -8.33 to -2.11; p < 0.001), fasting insulin (WMD: -1.32 µIU/mL; 95% CI -2.06 to -0.58; p < 0.001), HbA1c (WMD: -0.12 %; 95% CI -0.23 to -0.01; p = 0.04), and homeostatic model assessment of insulin resistance (HOMA-IR) (WMD: -0.69; 95% CI -1.00 to -0.38; p < 0.001) [149]. Similarly, an umbrella systematic review and meta-analysis (included eight meta-analyses of adults patients with various baseline health conditions) demonstrated that coenzyme Q10 significantly reduced fasting glucose levels (effect size (ES)_WMD_: -5.04 mg/dL; 95% CI -7.67 to -2.40; p < 0.001), insulin levels (ES_WMD_: -1.32 µIU/mL; 95% CI -2.06 to -0.58; p < 0.001), HbA1c (ES_WMD_: -0.17 %; 95% CI -0.32 to -0.01; p = 0.03), and HOMA-IR (ES_WMD_: -0.72; 95% CI -1.02 to -0.42; p < 0.001) [150].

Emerging evidence suggests that MVM supplementation may help mitigate the nutritional deficiencies observed in patients receiving glucagon-like peptide-1 receptor agonists, including those who have undergone bariatric surgery [151-153]. Accordingly, the integration of MVM supplementation alongside dietary management should be considered in patients using glucagon-like peptide-1 receptor agonists for obesity and type 2 diabetes [154]. However, current evidence remains limited, and further studies with standardized methodologies are needed to confirm these findings and strengthen clinical guidelines.

Cognition and Neurological Health

The impact of MVM supplementation on cognitive function has been an area of evolving research, with recent large-scale trials providing more definitive insights [20,24,155]. A meta-analysis of 10 RCTs involving 3,200 participants found that MVM supplementation enhanced immediate free recall memory but had no significant effect on delayed free recall memory or verbal fluency [156]. A pooled analysis of three COSMOS cognition sub-studies (COSMOS-Clinic, COSMOS-Mind, and COSMOS-Web), involving 5,203 non-overlapping participants, demonstrated beneficial improvements in both global cognition (mean difference (MD): 0.07 SU; 95% CI 0.03 to 0.11; p = 0.0009) and episodic memory (MD: 0.06 SU; 95% CI 0.03 to 0.10; p = 0.0007), with an effect equivalent to two years reduction in cognitive aging [24]. A meta-analysis of 28 RCTs showed that MVM supplementation significantly reduced Pittsburgh Sleep Quality Index scores (PSQI; MD: -0.70; 95% CI -1.37 to -0.03; p < 0.05), increased sleep efficiency (+2.58 min, 95% CI 2.01 to 3.16; p < 0.00001), prolonged total sleep time (SMD: 0.23; 95% CI 0.04 to 0.43; p < 0.05), and reduced sleep latency (SMD: -0.24; 95% CI -0.37 to -0.10; p < 0.001) and wake after sleep onset (SMD: -0.30; 95% CI -0.48 to -0.12; p < 0.001) [157].

In addition to MVMs, various nutraceuticals have shown promising results for cognitive enhancement. Multiple RCTs and meta-analyses have shown beneficial effects of ginseng in improving cognitive functions, daily activities, and overall quality of life [103,158,159]. Similarly, a meta-analysis of nine studies reported improved cognitive performance (MD: -17.9 ms; 95% CI -24.6 to -11.2; p < 0.001) with brahmi extract [160]. Ashwagandha supplementation (90 days) has also been associated with reduced perceived stress scale scores (19.5 ± 3.2 to 13.0 ± 5.0; p < 0.0001), improved PSQI scores (p < 0.0001), and decreased serum cortisol levels (9.04 ± 3.77 μg/dL to 6.34 ± 2.31 μg/dL; p < 0.0001) [161]. In addition, an RCT on L-theanine demonstrated improved sleep quality (p = 0.013) and enhanced cognitive functions such as verbal fluency (p = 0.001) and executive function (p = 0.031) [162]. Lutein and zeaxanthin supplementation have been associated with improvements in both visual and cognitive performance in adults and children [163,164]. Similarly, astaxanthin has shown cognitive benefits, as evidenced by a meta-analysis of clinical trials [165]. Furthermore, a meta-analysis including 120 pediatric patients and 226 adults demonstrated that coenzyme Q10 supplementation effectively reduced both the number of migraine days/month (p < 0.00001) and the duration of migraine episodes (p = 0.009) [166]. Together, these findings highlight the potential role of MVMs and nutraceuticals in supporting cognitive function and mitigating age-related cognitive decline.

Bone Health and Osteoporosis

Bone health is critically dependent on an adequate supply of various micronutrients, especially calcium and vitamin D, as they are essential for bone mineralization and the prevention of osteoporosis [26]. A meta-analysis of six RCTs (49,282 participants, 5,449 fractures, 730 hip fractures) reported that combined supplementation with vitamin D and calcium reduced the risk of any fracture by 6% (RR: 0.94; 95% CI 0.89 to 0.99) and hip fracture by 16% (RR: 0.84; 95% CI 0.72 to 0.97). However, vitamin D supplementation alone did not reduce fracture risk [26]. Similarly, a meta-analysis including 43,869 participants found that combined vitamin D and calcium supplementation significantly improved bone mineral density at the pelvis (SMD: 0.20; 95% CI 0.05 to 0.35; p = 0.01), with no statistically significant effect on overall fracture risk [167]. Beyond calcium and vitamin D, other components of MVM supplements, such as vitamin B-12, folic acid, vitamin C, vitamin K (especially vitamin K2-7), zinc, magnesium, and phosphorus, are also recognized for maintaining strong bones [25,168]. In an RCT conducted among Chinese adult men, six years of MVM supplementation led to a 63% reduction in fracture risk during the trial. Remarkably, this protective effect persisted and remained statistically significant (p = 0.02) up to 10 years post-intervention. However, no significant impact was observed in women throughout the study period [25]. In India, a study involving underprivileged premenarchal girls reported that calcium supplementation, with or without multivitamins and zinc, resulted in notable improvements in bone health [169]. However, direct evidence from RCTs evaluating their effects on bone health is lacking.

Immune Function and Susceptibility to Infection

It has been well-established that nutrients such as vitamin A, vitamin D, vitamin C, vitamin E, vitamin B-6, vitamin B-12, and minerals like zinc, iron, copper, and selenium play crucial roles in supporting the immune response [170]. In a 12-week randomized trial involving healthy adults aged 55-75 years, daily MVM supplementation improved plasma vitamin C (change from baseline +59.7 ± 6.4 µmol/L, p < 0.0001) and serum zinc (change from baseline +27.2 ± 3.7 µg/dL, p < 0.0001) levels and enhanced self-reported health status. However, it did not alter objective immune markers such as neutrophil activity or cytokine profiles [171]. Conversely, in nursing home residents, MVM supplementation showed no overall reduction in infections. However, a lower rate of infections was observed in certain subgroups, particularly those without dementia [172]. These findings suggest that while MVM supplementation may modestly improve micronutrient status, its effects on measurable immune cell function and infection prevention remain unconfirmed. Regarding nutraceuticals, an RCT reported an immunoenhancement effect of Korean red ginseng with an increase in T cells, B cells, and white blood cells [173].

Age-Related Eye Diseases

Antioxidant vitamin supplements have been suggested to decrease the risk of age-related cataracts [174]. A meta-analysis of 12 cohort studies and two RCTs reported that MVM supplementation modestly reduced the risk of nuclear cataracts [174]. Similarly, a large RCT (n = 14,641) in men physicians showed that long-term daily MVM supplementation significantly decreased the risk of cataract, although it had no significant effect on age-related macular degeneration (AMD) [175]. In this ancillary study of the COSMOS RCT, cocoa extract supplementation for a median of 3.6 years had no overall effect on the occurrence of AMD among older women and men [176]. The Age-Related Eye Disease study (AREDS) found that antioxidants (vitamin C, vitamin E, and vitamin A) and zinc significantly reduced the risk of AMD progression from intermediate to advanced [177]. Moreover, the five-year follow-up AREDS2 study reported a (HR: 0.90; 98.7% CI 0.76 to 1.07; p = 0.12) for lutein/zeaxanthin and (HR: 0.97; 98.7% CI 0.82 to 1.16; p = 0.70) for docosahexaenoic acid (DHA) + eicosapentaenoic acid (EPA), which did not significantly reduce AMD progression [178]. However, thereafter, a multicenter epidemiologic follow-up study of the AREDS2 clinical trial reported that, compared to vitamin A, lutein and zeaxanthin had a potential beneficial association with late AMD progression (HR: 0.85; 95% CI 0.73 to 0.98; p = 0.02) [179]. Together, these findings suggest that MVM supplementation, particularly when combined with specific nutraceuticals, may offer protective benefits for cataracts and AMD.

Infertility and Post-menopausal Symptoms

Human fertility is steadily declining due to various internal and external factors [33]. Though assisted reproductive technologies (ART) offer hope for couples facing infertility, achieving successful reproductive outcomes remains challenging [180]. In such a situation, nutrient supplementation represents a promising strategy for improving reproductive outcomes in women undergoing ART procedures [181]. Further, multivitamin use (≥6 times/week) was associated with a reduced risk of ovulatory infertility (multivariate adjusted relative risk: 0.59; 95% CI 0.46 to 0.75; p < 0.001), as reported by the large prospective cohort study (Nurses’ Health Study II), including 18,555 women and 26,971 eligible pregnancies, during eight years of follow-up [182]. A meta-analysis has shown a significant improvement in fertilization and pregnancy outcomes with omega-3 fatty acid supplementation in women who received fertility treatment [183]. Moreover, meta-analyses and cohort studies suggest that coenzyme Q10 supplementation, alone or in combination with vitamin C, vitamin E, vitamin B-9, vitamin B-12, L-carnitine, and zinc, improves sperm motility, concentration, quality, and other semen parameters; however, its effects on pregnancy and live birth rates remain inconclusive [32,33,184,185]. A meta-analysis on coenzyme Q10 supplementation and men infertility showed a significant effect on seminal concentration (RR: 49.55; 95% CI 46.44 to 52.66; p = 0.27), sperm concentration (RR: 5.33; 95% CI 4.18 to 6.47; p = 0.09), and sperm motility (RR: 4.50; 95% CI 3.92 to 5.08; p = 0.98) [185]. Similarly, alpha-lipoic acid has shown an improvement in sperm concentration (MD: 3.98; 95% CI 2.28 to 5.67; p < 0.00001), total sperm motility (MD: 6.68; 95% CI, 4.88 to 8.48; p < 0.00001), and semen volume (MD: -0.17; 95% CI -0.31 to -0.02; p = 0.03) [186]

Statin-Associated Muscle Symptoms

Several nutritional interventions have been explored as adjunctive strategies to manage statin-associated muscle symptoms (SAMS). Among micronutrients, vitamin D and coenzyme Q10 have been most frequently investigated for SAMS [187-189]. Evidence for coenzyme Q10 reported symptomatic improvement in muscle pain (WMD: -1.60; 95% CI -1.75 to -1.44; p < 0.001), muscle weakness (WMD: -2.28; 95% CI -2.79 to -1.77; p = 0.006), muscle cramps (WMD: -1.78; 95% CI -2.31 to -1.24; p < 0.001), and muscle tiredness (WMD: -1.75; 95% CI -2.31 to -1.19; p < 0.001) [190]. Similarly, a meta-analysis of seven RCTs reported that coenzyme Q10 supplementation was associated with an overall significant reduction in myopathic pain intensity (WMD: -0.96; 95% CI -1.88 to -0.03; p < 0.05) [191]. In an observational study of 106 patients with SAMS, coenzyme Q10 supplementation (50 mg twice daily) significantly reduced muscle pain after one month, with mean Visual Analogue Scale (VAS) scores of 1.06 ± 0.84 (p = 0.0001) and pain interference scores of 7.06 ± 0.79 [192].

Miscellaneous

Vitamins, minerals, and antioxidant-enriched oral nutritional supplements led to better wound healing rates following surgery, with notable benefits from vitamin C, vitamin D, vitamin E, and zinc for diabetic foot and pressure ulcers [193]. A long-term prospective study shows that nutritional supplementation helps maintain stable nutritional status after gastric bypass [194].

The role of MVM supplementation in specialized populations

Pregnancy and Lactation for Supporting Maternal and Fetal Health

A healthy and varied diet is essential for maternal nutrition; however, it may not fully meet the increased nutrient requirements necessary for maternal health and fetal development [195]. Hence, supplementation with key micronutrients, including iron and folic acid, is recommended to achieve positive pregnancy outcomes [29].

A meta-analysis of 20 trials involving 141,849 women compared MVM supplements (including iron and folic acid) against iron and/or folic acid alone. The findings support replacing iron and folic acid supplements with MVM for pregnant women, particularly in low‐ and middle‐income countries [29]. Consistent with these findings, an umbrella review of eight meta-analyses (including over three million mother-offspring pairs) found that prenatal folic acid and multivitamin supplementation were associated with a significantly reduced risk of autism spectrum disorder in offspring by 34% (RR: 0.66; 95% CI 0.55 to 0.80) [196]. Furthermore, an RCT including 194 women evaluated the efficacy of MVM supplementation with magnesium, which reported more effective outcomes in preeclampsia [197]. A meta-analysis of 15 RCTs showed that ≥90% adherence to MVM supplementation was associated with increased birthweight (MD: 18 g; 95% CI 3 to 33), reduced the risk of low birthweight (relative risk: 0.93; 95% CI 0.88 to 0.98) and small-for-gestational age (relative risk: 0.96; 95% CI 0.92 to 1.00) compared to iron-folic acid [198]. In contrast, an RCT of 3,332 pregnant women in rural Niger found no significant differences in birthweight, maternal weight gain, or anemia between MVM supplementation, lipid-based supplementation, and iron-folic acid, suggesting that additional or combined strategies may be required to improve pregnancy outcomes [199]. A meta-analysis of prenatal amino acid supplementation, particularly from the arginine family, demonstrated beneficial effects by improving fetal growth and birth weight in complicated pregnancies [200-202].

Similarly, a prospective RCT including 47 pregnant women reported a significantly increased maternal macular pigment optical volume (p < 0.001) and a 20% increase in infant macular pigment optical density (p = 0.242) with lutein and zeaxanthin supplementation [203]. These findings underscore the importance of integrating MVM supplementation into maternal health programs to improve outcomes for both mothers and their infants.

Infants, Children, and Adolescents for Growth and Development

Micronutrient deficiencies among infants and children are linked to a range of short- and long-term consequences, including physical, developmental, and cognitive delays, increased susceptibility to infections, higher morbidity and mortality rates, and reduced productivity later in life [30,31]. Because multiple deficiencies often occur simultaneously, MVM supplementation is generally considered more beneficial than addressing single deficiencies alone.

Evidence from meta-analyses and several clinical studies suggests that MVM supplementation in infants, children, and adolescents supports healthy growth and development by improving height, weight, motor and mental development, as well as linear and ponderal growth [204-207]. A previous RCT reported improved brain function in healthy children aged 8-14 years during 12 weeks of MVM supplementation [208]. A meta-analysis of 50 RCTs reported that MVM supplementation significantly increased hemoglobin levels (MD: 4.82 g/L; 95% CI 2.32 to 7.32) among patients aged 5 to 24 years with anemia and was also associated with modest improvements in height (MD: 0.87 cm; 95% CI 0.16 to 1.59) [209]. Nonetheless, studies evaluating the impact of MVM supplements on academic performance in healthy school children reported mixed outcomes, ranging from positive to marginal to no improvement [34,210,211].

Other nutraceuticals also play critical roles in child development. Choline and docosahexaenoic acid (DHA) are essential for brain and eye development, and recent findings highlight a synergistic relationship between choline and DHA [212]. Further, data from the Copenhagen school child intervention study showed a significant association between arginine intake and growth velocity (p = 0.04) [213]. Furthermore, a meta-analysis involving children under five years in low- and middle-income countries found no significant effect on stunting, wasting, and underweight, but reported 50% and 55% reduced risk of iron deficiency and iron-deficiency anemia, respectively [31]. Randomized trials in Palestine and Mexico reported improved growth in infants after 12 months of MVM supplementation [214,215].

Older Adults for Addressing Age-Related Nutritional Deficiencies

In older adults, adequate nutritional intake is essential to help prevent comorbidities such as increased susceptibility to acute and chronic illness, impaired immune function, cognitive decline, and malnutrition. However, age-related changes in digestion, absorption, and metabolism increase the need for nutrient-dense foods; hence, MVM supplementation is often recommended [216].

Previous studies in older adults (50-75 years) have shown that MVM supplementation improves micronutrient status (vitamin C, zinc, vitamin B-12, folate, and homocysteine), enhances self-reported health, and improves episodic memory, although no effects were observed on immune status or vitamin D levels [35,171]. However, another trial in older adults aged ≥65 years found no cognitive benefit [217]. Further, an RCT in older adults >70 years found that 12 weeks of MVM supplementation increased friendliness in females (p = 0.045) and reduced stress reactivity (p = 0.019) as well as loneliness in males (p = 0.042) [20]. In a COSMOS ancillary study including older adults (n = 958), the two-year effects of daily MVM supplementation along with cocoa flavanol were evaluated. The study reported modestly slowed biological aging, with a significant reduction in the rate of change for PCGrimAge (-0.113 years/year; 95% CI -0.205 to -0.020; p = 0.017) and PCPhenoAge (-0.214 years/year; 95% CI -0.410 to -0.019; p = 0.032), while cocoa extract showed no significant effect [218].

Athletes for Enhancing Physical Performance and Recovery

Adequate micronutrients may provide athletes with a competitive edge by enhancing overall training potential, which relies on a combination of proper nutrition and appropriate exercise [219].

In an RCT involving Chinese field artillery personnel, one week of MVM supplementation supported the recovery of psychological function, physical performance, and the neuroendocrine-immune system in young men consuming a typical Chinese diet [220]. Similarly, in well-trained endurance runners, MVM supplementation for 21 days prior to a race and for two days afterward did not attenuate the loss of contractile function post-running exercise, but it appeared to accelerate the recovery of maximal force capacity during the post-race period [36]. Collectively, these findings suggest beneficial effects of MVM supplementation in athletes by supporting recovery and helping restore peak physical performance after intense activity.

Safety, toxicity, and drug interaction

While MVM supplements may reduce the risks of certain chronic diseases, excessive use, particularly when the formulation is imbalanced, can potentially have harmful effects [221]. Hence, evaluating the safety of MVM supplements is as important as assessing their efficacy.

Among studies evaluating the efficacy of MVM supplements, there are a few reported safety outcomes or adverse events. Overall, the findings indicate that MVM supplements are well tolerated, with only minor adverse events reported across different populations. The Physicians’ Health Study II, which investigated the role of MVM supplements in cancer prevention in men with a median follow-up of 11.2 years, reported adverse events such as skin rashes and increased epistaxis [222]. Similarly, a systematic review of RCTs assessing the safety of MVM included nine studies in pregnant women and healthy adults, and six studies in the elderly, reported adverse events related to the gastrointestinal tract [221].

Drug-nutrient interactions can arise from physical, chemical, physiological, or pathophysiological mechanisms, and often include multiple overlapping elements [223]. The drugs can affect the efficacy of nutrients by impairing bioavailability or absorption, increasing intestinal or renal excretion, impairing micronutrient metabolism, and disrupting metabolic processes [224,225]. Although clinical evidence documents several common drug-nutrient interactions and their consequences [226,227], evidence specifically examining such interactions with multivitamin-multimineral (MVM) supplements remains limited. Hence, we are unable to summarize the potential drug-nutrient interactions in this review.

Key gaps and future directions

Despite substantial evidence supporting the role of MVM supplementation in chronic disease prevention, including special populations, significant research gaps remain. Existing studies vary widely in formulation, dosage, duration, and participant characteristics, limiting the generalizability of findings. Research on combined MVM-nutraceutical interventions is especially limited. Large, high-quality trials with standardized MVM formulations, including nutraceuticals, are needed to explore possible synergies with diet, lifestyle, and personalized strategies based on genetics, metabolism, and life stage. Evidence on MVM safety is also scarce, with few studies tracking adverse events. Additionally, most drug-nutrient interaction research focuses on single nutrients, leaving MVM-specific interactions largely unknown. Hence, long-term follow-up studies in diverse populations, including individuals with multiple health conditions or on polypharmacy, are required. Such evidence will be crucial for developing informed guidelines, improving formulations, and ensuring safe use in clinical settings.

Limitations

This narrative review has several limitations. The included evidence demonstrates substantial heterogeneity in MVM formulations, dosages, duration of supplementation, and study populations, which makes direct comparison and standardization of findings challenging. The lack of uniform definitions and composition of MVM products across studies further limits the ability to draw generalized conclusions. In addition, the RDAs and ULs referenced are based on ICMR-NIN 2024 guidelines; since these recommendations are region-specific and may not be fully applicable to populations with different dietary patterns, genetic backgrounds, or environmental exposures. Additionally, as a narrative review, the study may be subject to selection bias and does not include a formal systematic assessment of quantitative synthesis or risk of bias. As this was a narrative review, formal risk-of-bias assessment and quantitative synthesis were not performed. The review synthesizes findings from previously published studies without conducting any new meta-analysis or statistical pooling.

## Conclusions

MVM and nutraceuticals supplementation help prevent nutritional deficiencies and support overall health. Moreover, they are beneficial for cognitive performance, bone health, infertility, postmenopausal symptoms, and age-related eye conditions such as cataracts and AMD. However, evidence regarding the effects of MVMs on CVD, cancer, diabetes mellitus, and immune functions suggests variable outcomes, particularly in healthy populations. Further, MVMs appear safe for both short- and long-term use and are particularly valuable for populations with increased nutritional demands, including pregnant and lactating women, older adults, and athletes. Nutraceuticals such as omega-3 fatty acids, lutein, curcumin, coenzyme Q10, astaxanthin, and zeaxanthin provide targeted benefits that complement MVM supplements. Long-term studies involving diverse populations, individuals on polypharmacy, and combined use of MVMs with nutraceuticals are needed to better understand their efficacy and safety.
